# An Update on Targeted Treatment of IgA Nephropathy: An Autoimmune Perspective

**DOI:** 10.3389/fphar.2021.715253

**Published:** 2021-08-23

**Authors:** Xin Huang, Gaosi Xu

**Affiliations:** Department of Nephrology, The Second Affiliated Hospital of Nanchang University, Nanchang, China

**Keywords:** IgA nephropathy, targeted treatment strategies, autoimmune pathogenesis, renal inflammation, complement activation

## Abstract

Immunoglobulin (Ig) A nephropathy (IgAN) is the commonest form of primary glomerulonephritis worldwide and is, considered a significant cause of end-stage renal disease in young adults. The precise pathogenesis of IgAN is unclear. The clinical and pathological features vary significantly between individuals and races, which makes treating IgAN difficult. Currently, the therapeutic strategies in IgAN are still optimal blood pressure control and proteinuria remission to improve the renal function in most cases. Immunosuppressive drugs such as corticosteroids can be considered in patients with persistent proteinuria and a high risk of renal function decline; however, they include a high toxicity profile. Therefore, the safety and selectivity of medications are critical concerns in the treatment of IgAN. Various pharmacological therapeutic targets have emerged based on the evolving understanding of the autoimmune pathogenesis of IgAN, which involves the immune response, mucosal immunity, renal inflammation, complement activation, and autophagy; treatments based on these mechanisms have been explored in preclinical and clinical studies. This review summarizes the progress concerning targeted therapeutic strategies and the relevant autoimmune pathogenesis in IgAN.

## Introduction

Immunoglobulin (Ig) A nephropathy (IgAN), the commonest form of primary glomerulonephritis worldwide, manifests commonly as a progressive decline in renal function and results in high morbidity and mortality. IgAN accounts for approximately 10–20% of cases of primary glomerulonephritis in the United States; it has a higher prevalence, approximately 20–30%, in some European countries, with the highest prevalence of 40–50% in developed Asian countries (Lai et al., 2016; Schena and Nistor, 2018). Meanwhile, a cohort study from Sweden revealed that patients with IgAN have increased mortality—one extra death per 310 person-years—and a 6-years reduction in life expectancy, compared with matched controls (Jarrick et al., 2019). It is a significant cause of end-stage renal disease (ESRD) in young adults, and without effective intervention, IgAN causes irreversible worsening of renal function, thus resulting in the loss of labor force and a significant social burden (Schena, 2001; Floege and Amann, 2016).

**GRAPHICAL ABSTRACT:**
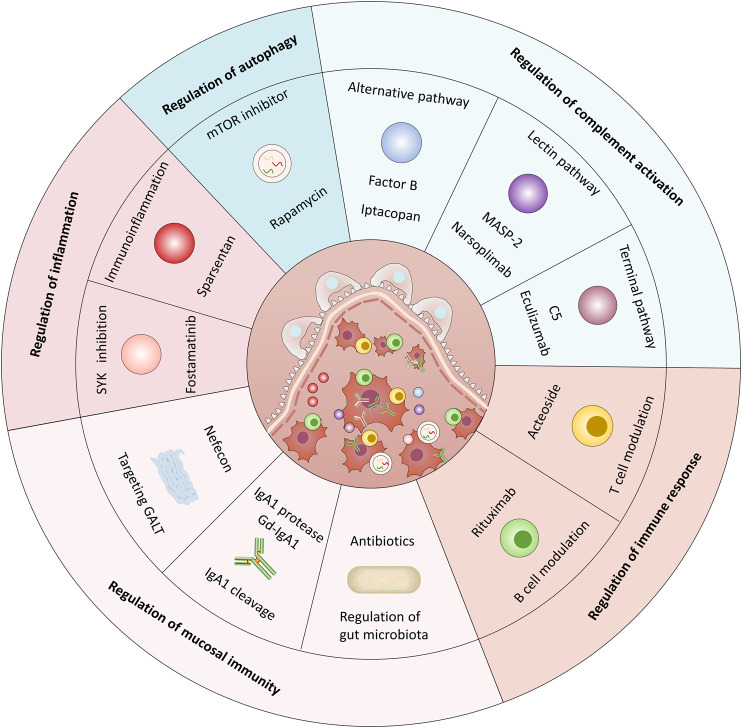


However, the precise pathogenesis of IgAN is not fully understood. Currently, interactions between genetic and environmental components are believed to determine the development of IgAN (Suzuki et al., 2011; Li et al., 2020). A well-accepted immunological pathogenesis is the multi-hit hypothesis. Abnormalities in the expression and activity of key glycosyltransferases involved in post-translational galactosylation in the Golgi apparatus result in reduced post-translational modified galactosylation of IgA1, which directly results in galactose-deficient IgA1 (Gd-IgA1) (Suzuki et al., 2009). Specifically, decreased expression and activity of core 1 β1,3-galactosyltransferase 1 and increased expression and activity of α-N-acetylgalactosaminide α2,6-sialyltransferase 2 are associated with increased Gd-IgA1 (Ju and Cummings, 2002; Suzuki et al., 2014). The overproduction of Gd-IgA1 as the first hit and a series of subsequent autoimmune responses, eventually lead to renal injury (Suzuki et al., 2011; Knoppova et al., 2016). The clinical and pathological manifestations of IgAN vary significantly between individuals and races. Therefore, the treatment options may differ between regions. For example, tonsillectomy has been recommended by some centers, particularly in Japan (Wyatt and Julian, 2013), which should not be performed as a treatment in Caucasian patients. Multiple studies from Japan have reported improved renal survival and partial or complete remission of hematuria and proteinuria following tonsillectomy with pulsed corticosteroids (Xie et al., 2003; Kawasaki et al., 2006; Komatsu et al., 2008; Kawamura et al., 2014).

Currently, the general therapeutic strategies in IgAN still focus on non-specific supportive treatment, including optimal blood pressure control using renin-angiotensin-aldosterone system (RAAS) blockers, and reduction of proteinuria (Chapter 10: Immunoglobuli, 2011). However, despite optimizing RAAS blockers, some patients cannot achieve effective alleviation of proteinuria and have a high risk of renal failure over time. The Kidney Disease: Improving Global Outcomes (KDIGO) clinical practice guidelines recommend a 6-months course of systemic corticosteroids for patients with persistent proteinuria (>1 g/d) despite 3–6 months of optimized RAAS blockade, and estimated glomerular filtration rate (eGFR) > 50 ml/min/1.73 m^2^; however, the risks and benefits of systemic corticosteroids should be weighed due to the increased risk of adverse events (AEs) (Chapter 10: Immunoglobuli, 2011). Additionly, the recent STOP-IgAN trial has questioned the efficacy of combined systemic immunosuppression and optimized RAAS blockade (Rauen et al., 2015). Therefore, novel treatments that target the pathogenic mechanisms in IgAN are urgently needed to arrest the disease progression. This review summarizes the emerging research on targeted therapeutic strategies and the relevant autoimmune pathogenesis in IgAN.

## Regulation of Immune Response in IgAN

### B-Cell Modulation

The involvement of a proliferation-inducing ligand (APRIL) and B-cell activation factor (BAFF) in the pathogenesis of IgAN has been the subject of extensive research. Given their essential roles in B-cell activation, targeting BAFF and APRIL in treating IgAN is a rational therapeutic approach.

Several studies have clarified the positive role of APRIL antagonism via reduced production of nephritogenic IgA in mouse models of IgAN. Compared with plasma levels of APRIL in healthy controls, increased levels have been reported in patients with IgAN which induce hyperproduction of Gd-IgA1 (Zhai et al., 2016; Muto et al., 2017; Takahara et al., 2019). However, decreased serum levels of IgA and reduced glomerular IgA deposition were observed in mice treated with anti-APRIL monoclonal antibodies, which effectively ameliorated the glomerular lesions defined by mesangial proliferation and sclerosis and subsequent albuminuria (Kim et al., 2015; Myette et al., 2019). VIS649 is a fully humanized monoclonal IgG2 antibody that targets and neutralizes human APRIL. Notably, a phase Ⅰ, randomized, placebo-controlled, single ascending dose trial (NCT03719443) to assess the safety and tolerability of VIS649 administered intravenously in healthy participants has been completed, and the preliminary results were presented by Suzuki et al. recently (Suzuki et al., 2021). The results suggested that a single dose of VIS649 was well-tolerated and safe in healthy adults, with no serious adverse events (SAEs) or AEs that led to study discontinuation. Serum IgA, Gd-IgA1, IgG, and IgM were reversibly suppressed in a dose-dependent manner with VIS649. Meanwhile, VIS649 was able to suppress free serum APRIL to the lower level of quantification. These data support the clinical development of VIS649 as a potential treatment for IgAN. Similarly, BION-1301, another novel humanized anti-APRIL monoclonal antibody, has been developed as a potential treatment for patients with IgAN. The interim results from phase I (NCT03945318) and phase II (NCT04684745) studies were presented by Barratt et al. recently and preliminary efficacy of BION-1301 was observed in patients with IgAN (Barratt et al., 2021a). The results revealed that BION-1301 was well-tolerated in patients with IgAN who received a 450 mg dose every 2 weeks for 12 weeks with no reported SAEs. A durable reduction in serum APRIL levels was also observed. Clinically meaningful reductions in proteinuria were observed as early as 12 weeks and were associated with a reduction in IgA levels.

BAFF, also known as B-lymphocyte stimulator (BLyS), is similar to APRIL in structure and function (Abrantes et al., 2020). Abnormalities in the production of BAFF are closely associated with B-cell-mediated autoimmune diseases, including IgAN. Serum levels of BAFF were reportedly significantly higher in patients with IgAN than those in controls; these levels were positively correlated with the severity of histopathological damage such as mesangial hypercellularity and segmental glomerulosclerosis, thus indicating that serum BAFF levels could be a noninvasive biomarker for monitoring the disease severity and a potential therapeutic target in IgAN (Xin et al., 2013; Li et al., 2014; Cao et al., 2020).

In light of the findings above, direct inhibition of BAFF alone or both BAFF and APRIL may be effective in inducing remission of IgAN by blocking their binding to the corresponding receptors and preventing the subsequent activation of downstream signaling pathways (Samy et al., 2017). Blisibimod, a monoclonal antibody against BAFF, is the first agent approved for the treatment of systemic lupus erythematosus, and clinical studies have evaluated its efficacy and safety in patients with IgAN (Lenert et al., 2017). A phase II/III study, BRIGHT-SC (NCT02062684), assessed the efficacy and safety of subcutaneous blisibimod in combination with standard therapy in patients with IgAN. The interim results were reported at ASN meetings (Kidney Week 2016); they suggested that B-cell subsets and Ig levels decreased significantly in the blisibimod group, thus demonstrating the pharmacological inhibition of BAFF. Meanwhile, a reduction in proteinuria was observed in the blisibimod group, while a steady increase was observed with placebo, which persisted until week 96 (% change from baseline −8.7 vs. 59.4%, *p* = 0.017). These data support the hypothesis that blisibimod-mediated BAFF inhibition reduces peripheral B-cells and immunoglobulins and may prevent a deterioration in the urine protein-to-creatinine ratio (UPCR) in patients with IgAN. Atacicept (NCT02808429 and NCT04716231) and RC-18 (NCT04291781) are recombinant human BLyS receptor-antibody fusion proteins that target both BAFF and APRIL, and phase II clinical trials to evaluate their efficacy and safety in the treatment of IgAN are currently ongoing (Table 1). Notably, the preliminary results of a phase II study (NCT02808429) examining the safety and efficacy of atacicept in reducing Gd-IgA1 and renal activity in patients with IgAN were previously presented by Barratt et al. recently (Barratt et al., 2020). The interim analysis revealed that, at week 24, patients with IgAN had a consistent, dose-dependent reduction in IgA, IgG, IgM, and Gd-IgA1, and a higher median % reduction from baseline in UPCR with atacicept than did those who received placebo. Meanwhile, the eGFR remained stable, and no SAEs were reported. These results suggest atacicept as a new treatment option in IgAN.

**TABLE 1:** Clinical trials on targeted drugs for IgAN.

Drug name (NCT number)	Drug target	Phase/Status	Inclusion criteria	Intervention/Treatment	Primary outcome measures (Time frame)
VIS649 (NCT03719443)	Anti-APRIL monoclonal antibody	I/completed	Healthy	VIS649 0.5 mg/kg-20 mg/kg, single dose, IV	Number of participants with TEAE (112 days) Frequency of 12-lead ECG treatment emergent abnormalities (112 days)
Blisibimod (NCT02062684)	Anti-BAFF monoclonal antibody	II/III completed	Proteinuria 1–6 g/d	Blisibimod, SC	Proportion of subjects achieving reduction in proteinuria from baseline (24 weeks)
Atacicept (NCT02808429)	Recombinant fusion protein against BAFF and APRIL	II/terminated	UPCR 0.75–6 g/gCr	Atacicept 25 mg or 75 mg, once weekly, SC, 72 weeks	Percentage of participants with AEs (96 weeks)
Atacicept (NCT04716231	Recombinant fusion protein against BAFF and APRIL	II/not recruiting	Urine protein excretion ≥1 g/d or UPCR ≥1 g/gCr, eGFR ≥45 ml/min/1.73 m^2^, SBP ≤150 mmHg and DBP ≤90 mmHg	Atacicept, once weekly, SC	Change from baseline in UPCR (24 weeks)
RC18 (NCT04291781)	Recombinant fusion protein against BAFF and APRIL	II/not recruiting	Urine protein excretion ≥1 g/d, eGFR >45 ml/min/1.73 m^2^	RC18 160 mg or 240 mg, once weekly, SC, 24 weeks	Change from baseline in 24 h urine protein excretion level (24 weeks)
Rituximab (NCT04525729)	Anti-CD20 monoclonal antibody	IV/recruiting	Proteinuria ≥1 g/d, eGFR >30 ml/min/1.73 m^2^, SBP <130 mmHg, DBP <80 mmHg, Serum albumin >25 g/L	Rituximab 1 g, Day 1 and Day 31, IV	Changes in proteinuria levels over 1 year compared with baseline (1 year)
Fostamatinib (NCT02112838)	SYK inhibitor	II/completed	Proteinuria >1 g/d at diagnosis and >0.5 g/d at the second screening visit	Fostamatinib 150 mg or 100 mg, twice daily, by mouth, 24 weeks	Mean change of proteinuria at week 24 (24 weeks)
LNP023 (NCT03373461)	Complement factor B inhibitor	II/not recruiting	Urine protein ≥1 g/d at screening and ≥0.75 g/d after the run-in period, eGFR ≥30 ml/min/1.73 m^2^	LNP023, twice daily, by mouth	Change from baseline of UPCR (90 days)
LNP023 (NCT04557462)	Complement factor B inhibitor	III/not recruiting	EGFR ≥20 ml/min/1.73 m^2^	Single arm, LNP023 200 mg, twice daily, by mouth	Number and percentage of patients with serious AEs, AEs, AEs of special interest, abnormalities in vital signs (Date of first administration to 7 days after the last administration)
LNP023 (NCT04578834)	Complement factor B inhibitor	III/recruiting	UPCR ≥1 g/gCr at screening and after the run-in period	LNP023 200 mg, twice daily, by mouth	Ratio to baseline in UPCR at 9 months (9 months) Annualized total eGFR slope over 24 months (24 months)
IONIS-FB-LRx (NCT04014335)	Antisense inhibitor of complement factor B	II/recruiting	Hematuria, Proteinuria	Single arm, IONIS-FB-LRx, Weeks 1, 3, 5, and every 4 weeks through Week 25, SC	Percent reduction in 24 h urine protein excretion (baseline to week 29)
APL-2 (NCT03453619)	Complement C3 inhibitor	II/not recruiting	UPCR >0.75 g/gCr, eGFR ≥30 ml/min/1.73 m^2^	Single arm, APL-2, once daily, SC, 48 weeks	Change from baseline of UPCR (48 weeks)
OMS721 (NCT03608033)	Humanized anti-MASP-2 monoclonal antibody	III/recruiting	Proteinuria >1 g/d or UPCR >0.75 g/gCr, eGFR ≥30 ml/min/1.73 m^2^ at screening and baseline	Narsoplimab (OMS721), 12 weeks	Change from baseline in 24 h urine protein excretion at 36 weeks (36 weeks)
Ravulizumab (NCT04564339)	Humanized anti-C5 monoclonal antibody	II/recruiting	Proteinuria ≥1 g/d	Ravulizumab, IV, 50 weeks	Percentage change in proteinuria from baseline to week 26 (26 weeks)
Cemdisiran (NCT03841448)	Synthetic RNAi targeting complement C5	II/recruiting	Hematuria, urine protein ≥1 g/d	Cemdisiran (ALN-CC5), SC	Percentage change from baseline in UPCR at week 32 (baseline to week 32)
CCX168 (NCT02384317)	C5aR antagonist	II/completed	UPCR >1 g/gCr, eGFR >60 ml/min/1.73 m^2^	Single arm, CCX168, twice daily, by mouth, 84 days	Number of patients with AEs (169 days)

APRIL, a proliferation-inducing ligand; BAFF, B cell activation factor; TEAE, treatment-emergent adverse events; IV, intravenous infusion; SC, subcutaneous injection; AEs, adverse events; ECG, electrocardiogram; UPCR, urine protein to creatinine ratio; eGFR, estimated glomerular filtration rate; SBP, systolic blood pressure; DBP, diastolic blood pressure; SYK, spleen tyrosine kinase.

Increased circulating levels of Gd-IgA1 and production of anti-Gd-IgA1 autoantibodies are two critical factors that contribute to the pathogenesis of IgAN. Therefore, depleting antibody-producing B-cells may be a potential therapy in IgAN because it would presumably reduce the production of pathogenic autoantibodies. B-cell depletion with the monoclonal antibody rituximab is well-tolerated and effective in several glomerular diseases. However, there is little evidence of its therapeutic potential in patients with IgAN (Jayne, 2010; Fervenza et al., 2019). On one hand, several case reports have demonstrated that rituximab could reduce albuminuria and improve renal function in patients with IgAN (Chancharoenthana et al., 2017; Lundberg et al., 2017). On the other hand, a small randomized, controlled trial in patients at risk of progressive IgAN reported that rituximab therapy failed to reduce albuminuria over 1 year and was associated with more AEs per patient. Additionally, serum levels of Gd-IgA1 and IgG anti-Gd-IgA1 autoantibodies did not reduce with rituximab treatment despite the effective depletion of B-cells (Lafayette et al., 2017). A possible reason for this observation is the prominent involvement of the mucosal, particularly intestinal, immune system in IgAN along with varying responsiveness of B-cells derived from different compartments to rituximab (Floege, 2017). Therefore, rituximab-resistant mucosal B-cells may contribute to the failure of rituximab in reducing the serum Gd-IgA1 level (Zhang and Zhang, 2018), which requires further investigation. Several hypotheses have been proposed regarding the origin of circulating polymeric Gd-IgA1 in IgAN. One possibility is that mucosal IgA1-secreting cells mimic the bone marrow or other systemic sites instead of homing to mucosal surfaces (Yeo et al., 2018). Another possibility is that of an enhanced mucosal IgA1 response that leads to a “spillover” of Gd-IgA1 into the circulation (Magistroni et al., 2015). Therefore, given the ineffectiveness of rituximab, mucosal-associated lymphoid tissues, the inductive sites of the originating B-cells, may be considered candidate tissues of Gd-IgA1 production, especially the Peyer’s patches, isolated lymphoid follicles and tonsils (Xie et al., 2004; Perše and Večerić-Haler, 2019). Notably, another randomized, controlled, single-blind study (NCT04525729) on rituximab in treating primary IgAN is now recruiting participants in China, and the results are expected in 2023.

### T-Cell Modulation

There is increasing evidence that T cells are associated with the initiation, progression, and disease severity in IgAN. However, the exact mechanisms are not fully understood (Tang et al., 2020). IgAN is characterized by higher proportions of circulatory Th2, Tfh, Th17, Th22, and γδ T-cells and lower proportions of Th1 and Treg cells (Ruszkowski et al., 2019). Hence, T-cell modulation may have renoprotective effects in the treatment of IgAN.

Th1/Th2 imbalance may induce the proliferation of B-lymphocytes and the production of abnormal IgA1 (Tang et al., 2020). Interleukin (IL)-4 (Th2-type), IL-17 (Th17-type), and IL-21 (Tfh-type) are believed to enhance Gd-IgA1 production (Ruszkowski et al., 2019). Meanwhile, IL-21 may be associated with the production of anti-Gd-IgA1 autoantibodies. Furthermore, IgAN involves a numerical and functional deficiency of Treg cells, which cannot inhibit abnormal immune responses (Ruszkowski et al., 2019). A combination of artemisinin and hydroxychloroquine (AH) therapy has demonstrated immunomodulatory effects in mouse models of IgAN. Studies have revealed that AH therapy improves renal function and decreases mesangial matrix expansion and immune complex deposition by reducing the proportions of Th2 and Th17 cells and promoting differentiation of Th1 and Treg cells (Bai et al., 2019a). Additionally, another study found that vitamin D3 had regulatory effects on the Th17/Treg balance and could decrease the level of albuminuria and urine erythrocytes in mouse models of IgAN (Zhang et al., 2014).

Furthermore, overexpression of the C-C motif chemokine ligand (CCL)20, CCL22, and CCL27 in mesangial cells was observed in patients with IgAN; these factors are chemotactic for Th22 cells via interactions with the C-C chemokine receptor (CCR)4, CCR6, and CCR10, respectively, which trigger Th22 cell infiltration and inflammatory injury in the kidney (Gan et al., 2018a). Acteoside, the main component of *Rehmannia glutinosa*, has been reported to have renoprotective functions in IgAN. A study has demonstrated that acteoside relieves albuminuria and reduces Th22 lymphocytosis in patients with IgAN (Gan et al., 2018b). Simultaneously, the *in vitro* study indicated that acteoside could alleviate mesangial cell inflammation by inhibiting Th22 cell chemotaxis and differentiation. Interestingly, a potential mechanism of losartan and glucocorticoids based on Th22 disorders in IgAN has been identified in mouse models of IgAN. Both losartan and dexamethasone can reduce the expression of CCR10, CCL27, IL-22, and Th22 infiltration in the kidney (Xiao et al., 2017). However, there are limitations in the scale and reproducibility of these experiments, and more animal and clinical studies are required to verify the efficacy and safety of these drugs.

## Regulation of Mucosal Immunity in IgAN

### Targeting Gut-Associated Lymphoid Tissue

Evidence suggests a critical role of gut-associated lymphoid tissue (GALT) as a potential source of poorly O-galactosylated IgA1 in IgAN (Coppo, 2018). Alimentary antigens or microorganism components or products initiate mucosal B cell activation and programming and IgA synthesis via T-cell-dependent or T-cell-independent mechanisms; among these, two events considered critical are the activation of the innate immune response—particularly through ligation of Toll-like receptors—and BAFF and APRIL signaling (Coppo, 2018; Yeo et al., 2018). Therefore, targeting dysregulated GALT immune responses may reduce Gd-IgA1 production, thereby improving IgAN. Nefecon, a novel enteric targeted-release formulation of budesonide (TRF-budesonide), releases the active compound in the ileocecal region where the Peyer’s patches are located to act locally by targeting mucosal immune dysfunction. A recent study in 16 patients explored the efficacy and safety of Nefecon in patients with IgAN (Smerud et al., 2011). In this study, 6 months of treatment with Nefecon 8 mg/day resulted in a significant reduction in albuminuria by 23% and an increase in eGFR by 8% without major corticosteroid-related side effects. NEFIGAN (NCT01738035) was a large randomized double-blind trial that included 150 patients with IgAN at risk of progression to ESRD, with eGFR of at least 45 ml/min/1.73 m^2^ and persistent proteinuria of at least 0.75 g/day despite optimized RAAS blockade. They were assigned randomly in a 1:1:1 ratio to 16 mg/day Nefecon, 8 mg/day Nefecon, or placebo groups (Fellström et al., 2017). Over 9 months, Nefecon (16 mg/day plus 8 mg/day) stabilized the eGFR and decreased the mean urine UPCR by 24.4% (−0.212 g/gCr) from the baseline value compared with an increase of 2.7% (0.024 g/gCr) with the placebo. The mean urine UPCR change from baseline was −27.3% in 48 patients who received 16 mg/day Nefecon and −21.5% in 51 patients who received 8 mg/day Nefecon. These effects were sustained throughout the 3 months follow-up. The incidence of AEs was similar across the treatment groups. However, 25/99 patients in the Nefecon group discontinued treatment or follow-up; of them, 16 patients did so due to AEs with systemic corticosteroid-related AEs being the commonest. This finding indicates a substantial systemic effect of TRF-budesonide and demonstrate that some amount of budesonide that is absorbed may act systemically rather than locally to attenuate albuminuria (Glassock, 2017). Additionally, a retrospective, propensity-matched study evaluated the efficacy of budenofalk, a controlled-release formulation of budesonide, in 18 patients with IgAN in comparison with systemic corticosteroids (Ismail et al., 2020). These results were consistent with the two aforementioned trials concerning albuminuria and renal function. Therefore, targeted release of budesonide could be a safe and practical approach in the treatment of patients with IgAN. Notably, the NefIgArd trial (NCT03643965) is a phase III, randomized, double-blind, placebo-controlled study to evaluate the efficacy and safety of Nefecon 16 mg/day in patients with primary IgAN at risk of progression to ESRD that included two parts. Part A included 9 months of treatment and 3 months of follow-up, and the primary outcome included the effects of Nefecon 16 mg on 24 h UPCR at 9 months. Part B included 12 months of follow-up without treatment, and the primary outcome included the effects on a 2-years eGFR-based endpoint. Barratt et al. presented the preliminary results of this phase III study recently (Barratt et al., 2021b). The geometric mean UPCR reduced by 27% in the Nefecon group compared with that in the placebo group (*p* = 0.0005) at 9 months. Meanwhile, compared with the placebo group at 9 months, the Nefecon group had statistically significant (7%; 3.87 ml/min/1.73 m^2^) treatment benefit on eGFR (*p* = 0.0029). There was a similar overall incidence of AEs between the two treatment groups. These results are indicative of a clinically meaningful reduced risk of future progression to ESRD in patients with IgAN treated with Nefecon.

### Inhibition and/or Cleavage of IgA1

Given that mucosal IgA is polymeric, the serum and mesangial Gd-IgA1 in patients with IgAN may be a typical form of mucosal IgA (Boyd et al., 2012). Immune complexes containing IgA1 are trapped by the transferrin receptor—which is overexpressed on mesangial cells—and the enzyme transglutaminase 2, thus resulting in mesangial cell activation and glomerular lesions (Lechner et al., 2016a). Therefore, it is sensible to formulate drugs that inhibit dysregulated mucosal immunity or promote the degradation of pathogenic IgA1.

Bacterial IgA proteases demonstrate substrate specificity and attacking the hinge region of IgA1 (Xie et al., 2010). Several studies have verified the ability of bacterial IgA proteases to degrade complex human IgA1 both *in vitro* and *in vivo* (Lamm et al., 2008; Wang et al., 2016). In one study, intravenous injection of IgA protease significantly removed both the antigen and antibody components in the glomeruli of an IgAN model (Lamm et al., 2008). Additionally, markedly diminished IgA1 mesangial deposits, glomerular inflammation, fibrosis, and hematuria were observed after injecting IgA1 protease in IgAN. The administration of IgA1 protease was also associated with a decrease in IgA1-soluble CD89 (sCD89) complexes, transferrin receptor, and transglutaminase-2 (Lechner et al., 2016b). sCD89 is believed to be involved in the formation of Gd-IgA1-containing complexes and promotion of the disease (Launay et al., 2000; Berthelot et al., 2012). Specifically, results of human studies have revealed that patients with stable clinical courses have high levels of sCD89, which is in contrast with the low levels observed in patients with progressive IgAN (Vuong et al., 2010). This finding indicates that the binding of sCD89 to polymeric Gd-IgA1 may have a protective effect. Furthermore, studies in animal models have suggested that treatment with IgA1 protease significantly reduced IgA1–sCD89 complexes and restored CD89 expression (Lechner et al., 2016b). Therefore, IgA1 protease may alleviate glomerular injury by reducing the deposition of Gd-IgA1-containing complexes in IgAN. Further investigations are needed for its application in patients with IgAN.

### Regulation of Gut Microbiota

The potential roles of intestinal microbiota in the relationship between the gut and the renal system in IgAN have garnered progressive attention recently (Coppo, 2018). A recent genome-wide association study of the gut microbiota identified nine genetic variants associated with IgAN susceptibility (He et al., 2021). These variants of gut microbiota were associated with sub-phenotypes of IgAN, i.e., early age at onset, elevated Gd-IgA1 levels, severe hematuria, and advanced chronic kidney disease (CKD) stage. It has been reported that the development of IgA-associated nephropathy in BAFF-Tg mice is dependent on the commensal flora, and elevated serum IgA phenotype and renal IgA deposition are ablated in these transgenic mice under germ-free conditions (McCarthy et al., 2011). Several studies have observed gut microbiota dysbiosis in patients with IgAN compared with healthy controls (De Angelis et al., 2014; Dong et al., 2020). Patients with IgAN have lower microbial diversity with increased pathogenic bacteria, such as *Escherichia* and *Shigella*, and reduced numbers of beneficial bacteria, such as *Bifidobacterium* (De Angelis et al., 2014; Hu et al., 2020; Zhong et al., 2020). Additionally, modifications of the gut microbiota are closely related to the clinical features in IgAN (Zhong et al., 2020). These findings indicate that regulation of gut microbiota and restoration of gut symbiosis may be beneficial in patients with IgAN.

A recent study successfully determined the therapeutic effects of oral antibiotics on the microbiota in a humanized mouse model of IgAN (Chemouny et al., 2019). Treatment with antibiotics significantly depleted the fecal microbiota and ameliorated IgA1 mesangial deposition, glomerular inflammation, and the severity of albuminuria. Accordingly, these effects were correlated with decreased levels of circulating IgA1-IgG complexes irrespective of the unaffected serum levels of IgA1 and IgG. Similarly, another study evaluated the therapeutic potential of rifaximin, a non-absorbable oral antibiotic, in IgAN (Di Leo et al., 2021). The results revealed that rifaximin reduced the UPCR, serum levels of IgA1-sCD89 and murine IgG-IgA1 complexes, and glomerular deposition of IgA1, thus indicating its possible role in the treatment of IgAN by regulating the gut microbiota. The proposed mechanism of rifaximin is via the restoration of symbiosis (i.e., increased *Bacteroidetes/Firmicutes* ratio) and binding to the pregnane X receptor, thereby promoting the restoration of the intestinal barrier function, inhibiting TLR-4/NF-kB signaling pathway, and decreasing the synthesis of tumor necrosis factor (TNF)-α. Reduction of TNF-α levels results in the downregulation of the polymeric immunoglobulin receptor, BAFF, and consequently Gd-IgA1 (Di Leo et al., 2021).

## Regulation of Inflammation in IgAN

### Inhibition of Spleen Tyrosine Kinase Activation

SYK is a cytosolic non-receptor protein tyrosine kinase that is highly expressed in the hematopoietic cells, primarily B-cells and myeloid cells (Kaur et al., 2013; McAdoo and Tam, 2018). SYK has recently been reported to play a pathogenic role not only in glomerular lesions but also tubulointerstitial injury in IgAN (McAdoo et al., 2015; Yiu et al., 2021). A study reported that pharmacological inhibition of SYK significantly reduced the production of proinflammatory cytokines such as IL-6 and IL-8, as well as the proliferation of human mesangial cells induced by IgA1, which are well-accepted events in the initiation of glomerular inflammation in patients with IgAN (Kim et al., 2012a). Furthermore, inhibition of SYK can alleviate inflammatory responses in activated tubular epithelial cells evoked by glomerulotubular crosstalk in IgAN via suppression of downstream nuclear factor (NF)-κB and p42/p44 mitogen-activated protein kinase (MAPK) pathways (Yiu et al., 2021).

Nevertheless, recent research on the effects of SYK inhibitors in IgAN is limited to the cellular level, and further studies in mouse models of IgAN are warranted to elucidate its precise function and mechanism of action. Fostamatinib disodium (R788), a prodrug SYK inhibitor, is converted by intestinal alkaline phosphatase to the active metabolite R406, which binds to the catalytic domain of SYK and, possibly exerts anti-inflammatory effects in IgAN. Notably, a phase II, randomized, double-blind, placebo-controlled clinical study (NCT02112838) to examine the safety and efficacy of fostamatinib in IgAN has been completed, and the results were reported recently (Tam Wk et al., 2019). Both doses of fostamatinib (100 and 150 mg) reduced proteinuria (spot protein-creatinine ratio, sPCR) compared to the baseline values, although the differences were not significant. In a pre-specified subgroup analysis of patients with baseline sPCR ≥1,000 mg/gCr, there was a dose-dependent reduction in sPCR, which did not reach statistical significance. There were no significant changes in the eGFR during the study period. Eight SAEs were observed in six patients in the treatment group (*n* = 51). These results suggest that further investigations of fostamatinib are warranted in patients with IgAN.

### Regulation of Inflammation-Related Signaling Pathways

**FIGURE 1:**
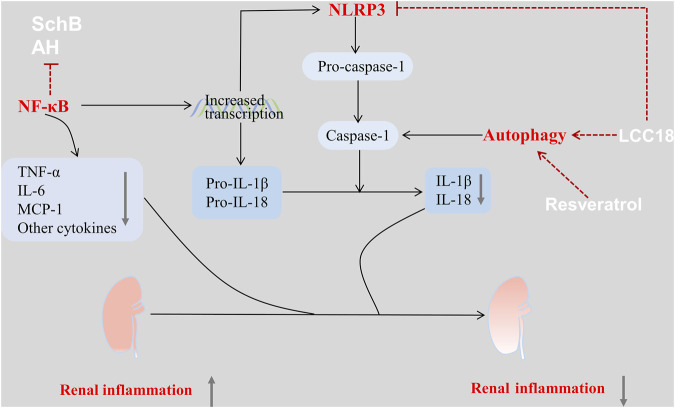
Drugs used in the treatment of IgAN via regulation of the inflammation-related signaling pathways. There are several inflammation-related pathways involved in the renal inflammation of IgAN, which can be used as pharmacological targets. The NLRP3 inflammasome plays a vital role among these pathways and can be activated by triggering activation of the NF-κB. Some drugs such as SchB, AH, LCC18 can act directly on the NLRP3 inflammasome or its upstream regulatory signaling, thus suppressing the proinflammatory pathways, decreasing the expression of inflammatory cytokines, and attenuating infiltration by inflammatory cells. These effects improve the renal pathology and kidney function in IgAN. Additionally, drugs such as LCC18, and resveratrol can also enhance autophagy, thereby inhibiting the NLRP3 inflammasome. Therefore, these drugs can exert a renoprotective effect in IgAN.

Nucleotide-binding oligomerization domain (NOD)-like receptor family pyrin domain containing 3 (NLRP3) inflammasome is a complex of cytosolic proteins that typically consists of NLRP3 protein, apoptosis-associated speck-like protein containing a CARD (ASC), and caspase-1. The pathogenic role of NLRP3 inflammasome in IgAN has recently been clarified (Hua et al., 2013). Additionally, IgA immune complexes (IgA-Ics) are suggested to serve as the priming signals for the NLRP3 inflammasome (Tsai et al., 2017; Peng et al., 2019). The assembly of the NLRP3 inflammasome leads to the activation of caspase-1 and activated caspase-1 is essential for cleaving pro-IL-1β and pro-IL-18 into IL-1β and IL-18, respectively (Chang et al., 2014). NF-κB signaling is a known critical signaling pathway in the renal inflammation of IgAN (Figure 1). Activation of NF-κB can induce the synthesis of inflammatory cytokines, such as TNF-α, IL-6, and MCP-1, in the pathogenesis of IgAN. Furthermore, activated NF-κB acts as the primary signaling for increased pro-IL-1β, pro-IL-18, and NLRP3 transcription, thereby activating the NLRP3 inflammasome (Chang et al., 2014).

Schisandrin B (SchB), the active compound of the Chinese herb *Schisandra chinensis*, can ameliorate renal function and attenuate mesangial cell proliferation as well as inflammatory cell infiltration in mouse models of IgAN. These anti-inflammatory effects are linked to the direct inactivation of the NF-κB signaling pathway in IgAN (Qin et al., 2019).

Hydroxychloroquine (HCQ) is a classical antimalarial drug with anti-inflammatory properties and is used to treat autoimmune diseases, such as rheumatoid arthritis and systemic lupus erythematosus (Nirk et al., 2020; Schrezenmeier and Dörner, 2020). The therapeutic potential of HCQ has been explored in patients with IgAN. Specifically, combined HCQ and RAAS inhibition significantly reduced proteinuria in patients with IgAN without evidence of AEs (Gao et al., 2017; Yang et al., 2018; Liu et al., 2019). However, the specific therapeutic mechanism of HCQ in IgAN remains unclear. Preclinical studies have indicated that AH therapy could improve kidney dysfunction and renal histological lesions in mouse models of IgAN via suppressed activation of the NF-κB/NLRP3 pathway (Bai et al., 2019b), thus suggesting powerful anti-inflammatory properties induced by AH via the NF-κB/NLRP3 pathway.

### Others

Endothelin-1 (ET-1) is strongly involved in renal cell injury, proteinuria, inflammation, and fibrosis resulting in CKD, mainly through the activation of endothelin A receptors (Kohan and Barton, 2014). ET-1 increases the formation of angiotensin II, which activates renal ET-1 production. Preclinical data have demonstrated that both endothelin and angiotensin II damage podocytes, and blockade of both pathways in models of CKD alleviated proteinuria and renal inflammation, protected the podocytes and prevented glomerulosclerosis (Komers and Plotkin, 2016).

Sparsentan is a first-in-class, orally active, dual-acting antagonist of endothelin type A (ETA) and angiotensin II type 1 (AT1) receptors (Komers et al., 2017). It is currently under evaluation in advanced phase III trials in the treatment of focal segmental glomerulosclerosis (FSGS) and IgAN. Results from a phase II (NCT01613118), randomized, double-blind, and active-control study demonstrated that patients with FSGS tolerated it well and achieved significantly greater reductions in proteinuria after 8 weeks of sparsentan compared to those who received irbesartan (Trachtman et al., 2018). Notably, the PROTECT study (NCT03762850), a phase III, randomized, double-blind, active-control study, is ongoing to determine the long-term nephroprotective potential of sparsentan in comparison with an angiotensin receptor blocker in patients with IgAN. Approximately 380 patients at high risk of disease progression despite optimized RAAS blockade will be randomly assigned in a 1:1 ratio to either the sparsentan or irbesartan arms in the study. The primary outcome is the change in UPCR from baseline at week 36.

## Regulation of Complement Activation in IgAN

### Inhibition of the Alternative Pathway

The complement system is an important component of the innate immune system. One study has highlighted the involvement of the alternative, lectin, and terminal pathways of complement activation in glomerular inflammation and injury in IgAN (Tortajada et al., 2019). The pathways of complement activation have been reported in detail previously (Walport, 2001a; Walport, 2001b). Additionally, mesangial C3 deposition and decreased serum C3 levels are associated with disease progression and poor kidney outcomes in patients with IgAN (Wyatt and Julian, 1988; Kim et al., 2012b; Nam et al., 2020). Notably, local synthesis and activation of C3 in resident glomerular cells were observed in patients with IgAN (Abe et al., 2001), which is consistent with the finding that Gd-IgA1 can induce C3 secretion from human mesangial cells *in vitro* (Schmitt et al., 2014).

Targeting alternative pathways to treat IgAN is currently under investigation. Iptacopan (LNP023) is an oral, selective, and reversible factor B inhibitor. Several clinical trials (NCT03373461, NCT04557462, and NCT04578834) have evaluated the safety, tolerability, and efficacy of LNP023 in patients with primary IgAN. Notably, Barratt et al. recently presented the results of the latest phase II clinical trial of LNP023 (NCT03373461) (Perkovic et al., 2021). An interim analysis after 90 days of treatment revealed that patients in the LNP023 group had a 23% reduction in urine protein compared with the placebo group (*p* = 0.038). Meanwhile, patients treated with LNP023 had little change in eGFR, which contrasted with a mean decrease in eGFR of 3.3 ml/min/1.73 m^2^ in the placebo group. There was no difference in the dose-dependent incidence of AEs between the two groups. A phase III study APPLAUSE (NCT04578834) is currently underway to investigate whether LNP023 could delay disease progression and improve clinical outcomes. IONIS-FB-LRx, an antisense inhibitor of factor B messenger ribonucleic acid, was developed to reduce the production of factor B in the liver. A phase II, single-arm open-label clinical study (NCT04014335) was designed to evaluate the efficacy and safety of IONIS-FB-LRx in adults with primary IgAN. Additionally, APL-2, a derivative of compstatin, is an inhibitor of C3 activation and can block the cleavage of C3 into C3a and C3b. A phase II study (NCT03453619) is ongoing for APL-2 in patients with IgAN, lupus nephritis, primary membranous nephropathy, or C3 glomerulopathy.

### Inhibition of the Lectin Pathway

In the pathogenesis of IgAN, it has been proposed that polymeric IgA1 may activate both the alternative and lectin pathways, while exposure to N-acetylgalactosamine of Gd-IgA1 may be capable of activating the lectin pathway (Medjeral-Thomas and O'Shaughnessy, 2020). Significant glomerular deposition of mannan-binding lectin (MBL), L-ficolin, MBL-associated serine proteases (MASPs), and C4d was observed in patients with IgAN, which indicates potential activation of the lectin pathway in the glomeruli (Endo et al., 1998; Roos et al., 2006). These results indicate that activation of the lectin pathway may play a critical role in IgAN.

Narsoplimab (OMS721) is a humanized monoclonal antibody against MASP-2, the critical effector enzyme of the lectin pathway. An interim analysis from a phase II study (NCT02682407) revealed that OMS721 treatment was well-tolerated and correlated with a substantial (61.4%) reduction in 24 h albuminuria excretion and a stable eGFR at 31–54 weeks of treatment, in eight patients with advanced IgAN (Lafayette et al., 2020). Most AEs were mild or moderate and transient. No treatment-related SAEs have been reported to date. Additionally, the efficacy of OMS721 is being evaluated in patients with IgAN with albuminuria > 1 g/d in a phase III, double-blind, randomized, placebo-controlled study (NCT03608033); the results are expected in 2023.

### Inhibition of the Terminal Pathway

All three complement activation pathways activate the terminal pathway, which includes cleavage of C5 into C5a and C5b by C5 convertase and subsequent formation of a membrane attack complex (MAC) consisting of C5b-9. The deposition of C5b-9 in the mesangium has been reported in patients with IgAN (Rauterberg et al., 1987). C5a is an anaphylatoxin, which interacts with C5aR to induce an exaggerated inflammatory response. It has been reported that urinary and renal C5a and renal expression of C5aR are positively correlated with the activity and severity of renal injury in patients with IgAN (Liu et al., 2014). Additionally, C5aR deficiency has been reported to significantly reduce albuminuria, mesangial proliferation, and histological damage in mouse models of IgAN (Zhang et al., 2017b). Therefore, targeting the components of the terminal complement pathway may be a viable option in IgAN via suppression of complement-dependent inflammation in the kidney.

**FIGURE 2:**
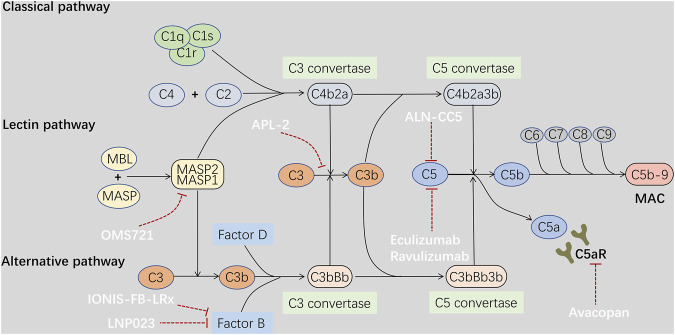
Drugs treat IgAN through regulation of complement activation. Complement activation through any of the three pathways ends up in activation of the terminal pathway, followed by cleavage of C5 by a C5 convertase, thereby leading to the release of C5a and formation of a membrane attack complex C5b-9. Circulating and mesangial polymeric IgA1 may activate both the alternative and lectin pathway systemically or locally in the glomeruli, resulting in deposition of complement fragments and glomerular inflammation in IgAN. C5a is an anaphylatoxin, which can induce an exaggerated inflammatory response through interaction with C5aR. In addition, C5b-9 deposition may stimulate inflammatory cytokines production by mesangial cells. Therefore, drugs targeting the alternative pathway Factor B and C3 such as LNP023, IONIS-FB-LRx, APL2, and the lectin pathway MASP2 such as OMS721, and the terminal pathway C5 and C5aR such as ALN-CC5, Eculizumab, Ravulizumab, Avacopan may be beneficial to IgAN through suppression of complement-dependent inflammation.

Eculizumab, a recombinant humanized monoclonal antibody selectively against C5, can inhibit C5 cleavage, thereby reducing the release of C5a and the formation of MAC (Figure 2). Beneficial effects, such as temporary stabilization of the renal function or reduction in albuminuria, have been observed with eculizumab in progressive IgAN (Rosenblad et al., 2014; Ring et al., 2015). Ravulizumab, the first long-acting complement inhibitor, has similar effects to eculizumab via C5 antagonism. Ravulizumab in IgAN is currently under evaluation in preclinical studies (McKeage and Duggan, 2019). Additionally, a phase II, double-blind, randomized, placebo-controlled study (NCT04564339) to evaluate the efficacy and safety of ravulizumab in patients with proliferative lupus nephritis or IgAN is now recruiting participants, and the results of this study will inform us of the efficacy of terminal complement inhibition in IgAN. Furthermore, cemdisiram (ALN-CC5), a synthetic RNAi that can inhibit C5 production in the liver, is also being tested in a phase II study in patients of IgAN at high risk of progression (NCT03841448). Avacopan (CCX168), is an orally active small molecule that selectively antagonizes C5aR, and its efficacy in patients with IgAN on stable RAAS blockade has been evaluated (NCT02384317) (Table 1). Based on these findings, regulation of complement activation may be a target in the treatment of IgAN.

## Regulation of Autophagy in IgAN

Autophagy is a highly conserved intracellular catabolic process that degrades damaged or superfluous organelles and biological macromolecules, and is critical for the maintenance of homeostasis in several cell types (Bhatia and Choi, 2020). The formation and fusion of autophagosomes with lysosomes are the two major steps in autophagy. Studies have identified decreased autophagy in the podocytes of patients with IgAN (Liang et al., 2016; Mao et al., 2019), which is associated with podocyte cell injury, death, dysregulated function, and ultimately proteinuria (Ye et al., 2019). Additionally, the inhibition of podocyte autophagy in IgAN may be induced by TGF-β1 secreted from the proliferating mesangial cells via the activation of the mammalian target of rapamycin complex 1 (mTORC1) pathway (Mao et al., 2019). Furthermore, mesangial cell proliferation in IgAN has also been reported to be partially attributed to activated mTORC1 and the resultant inhibition of autophagy (Xia et al., 2020). In contrast, autophagy can restrict inflammation by interacting with specific signaling pathways and engulfing inflammation triggers (Ye et al., 2019). Therefore, upregulation of autophagy may be beneficial in the treatment of IgAN

Rapamycin, an mTOR inhibitor, has been reported to enhance autophagy in mesangial cells in a mouse model of IgAN, thus decreasing proteinuria and attenuating mesangial matrix accumulation and cellular proliferation (Liu et al., 2017). Additionally, *in vitro* studies have demonstrated that rapamycin alleviated impaired autophagy in podocytes under stimulated conditions of IgAN, thereby reducing podocyte apoptosis (Liang et al., 2017). These results indicate that inducing autophagy by targeting mTORC1 may exert a cytoprotective effect on both the mesangial cells and podocytes in IgAN. Additionally, LCC18 (Yang et al., 2020) and resveratrol (Chang et al., 2015) have also been reported to improve renal inflammation via autophagy-mediated NLRP3 inflammasome inhibition in mouse models of IgAN. Autophagy is a dynamic and sophisticated process. It plays different roles in different cells, and excessive autophagy may be harmful (Sato et al., 2009). Therefore, it is necessary to clarify the specific role of autophagy in the pathogenesis of IgAN, to regulate autophagy and exert its therapeutic benefits more precisely.

## Conclusion and Prospects

In summary, the etiology of IgAN is multifactorial, and the clinical and histopathological features vary. Increased circulating levels of Gd-IgA1 and mesangial deposition of immune complexes containing IgA are key events in the pathogenesis of IgAN. Therefore, the ideal drug should specifically inhibit the production of pathogenic IgA1 and decrease mesangial IgA deposition to prevent or delay the progression of IgAN. Emerging factors such as BAFF, APRIL, B- cells, and dysregulated mucosal immunity have been demonstrated to be directly or indirectly involved in these pathogenic processes. Additionally, targeting the detrimental downstream consequences of IgA deposition such as complement activation and excessive renal inflammation have also been summarized. Notably, autophagy has been found to participate in the pathogenesis of IgAN but its specific role remains to be clarified. In this review, we summarized several drugs that target the autoimmune pathogenic mechanisms and are beneficial in IgAN. However, most of them are still in the preclinical stages, and more basic and clinical studies are urgently needed to advance their future application.
